# Acute Necrotizing Pancreatitis Complicated by Multiple Splanchnic Venous Thromboses and Bilateral Renal Infarctions in a Patient With Recent COVID-19 Infection: A Case Report

**DOI:** 10.7759/cureus.28049

**Published:** 2022-08-16

**Authors:** Wan Fen Chan, Yen Ni Toh

**Affiliations:** 1 Department of Anaesthesia & Surgical Intensive Care, Changi General Hospital, Singapore, SGP

**Keywords:** acute necrotizing pancreatitis, coronavirus disease 2019, case report, covid-19, vascular complications, renal infarction, splanchnic vein thrombosis, acute pancreatitis

## Abstract

Splanchnic vein thrombosis (SVT) is a well-known complication of pancreatitis, but extra-splanchnic thrombosis is rarely seen. We report a case of acute necrotizing pancreatitis complicated by portal vein thrombosis and resultant hepatic infarction, splenic vein thrombosis, bilateral renal infarction, and bowel hypoperfusion in an 81-year-old man with recent coronavirus disease 2019 (COVID-19) infection. To the best of our knowledge, this is the first documented case of such extensive intra-abdominal thromboses complicating severe acute pancreatitis. Despite multi-organ support and systemic anticoagulation, he deteriorated into multiple organ failure and died after 72 hours. He had no prior history of thrombotic disorders. COVID-19 infection can cause sustained prothrombotic changes, while severe acute pancreatitis also produces an inflammatory response that promotes coagulation. Together, the two concurrent disease processes may have resulted in the particularly extensive intra-abdominal thromboses and infarctions seen in this patient. Physicians should be mindful of the elevated risk of severe vascular complications in acute pancreatitis patients with concurrent or recent COVID-19 infection.

## Introduction

Splanchnic vein thrombosis (SVT) is a well-known complication of acute pancreatitis. Extra-splanchnic thrombosis, on the other hand, is rarely seen. Coronavirus disease 2019 (COVID-19) primarily affects the respiratory system, but it also has several extrapulmonary manifestations including hypercoagulability [1]. We report an unusual case of acute pancreatitis complicated by multiple splanchnic thromboses and bilateral renal infarctions. The patient’s recent COVID-19 infection may have contributed to a hypercoagulable state resulting in multiple thromboses. This case report was prepared following the CARE (CAse REports) guidelines [2].

## Case presentation

An 81-year-old Chinese man presented to our emergency department with a one-day history of nausea, vomiting, and acute epigastric pain radiating to his right shoulder. He had tested positive for COVID-19 infection 20 days prior, but only had mild symptoms. No history of previous similar episodes, travel, alcohol consumption, or recent trauma was reported. He had a history of hypertension, hyperlipidaemia, type 2 diabetes, Kidney Disease: Improving Global Outcomes (KDIGO) G3aA2 chronic kidney disease, and ischaemic heart disease, but no history of gallstone disease, deep vein thrombosis, or pulmonary embolism. The patient was haemodynamically stable, and physical examination was only remarkable for epigastric tenderness. Laboratory tests were significant for elevated lipase levels and leukocytosis. Of note, the platelet count and coagulation profile were normal. Values of his laboratory data have been summarized in Table 1.

**Table 1:** Laboratory data on admission * SI unit conversion: to convert PO_2_ to kPa, multiply by 0.133.

Variable	Result	Reference range
Leukocyte count (x 10^3^/µL)	23.2	4.0-10.0
Platelet count (x 10^3^/µL)	239	150-450
Urea, serum (mmol/L)	6.5	2.8-7.7
Glucose, serum (mmol/L)	10.9	3.1-7.8
Calcium, serum (mmol/L)	2.20	2.10-2.60
Albumin, serum (g/L)	45	37-51
Total bilirubin, serum (µmol/L)	9.4	5.0-30.0
Alanine transaminase, serum (U/L)	18	10-55
Aspartate transaminase, serum (U/L)	21	10-45
Lactate dehydrogenase, serum (U/L)	149	90-190
Partial pressure of oxygen (PO_2_) (mmHg*)	85.6	75.0-100.0
Prothrombin time (s)	10.5	9.5-11.5
Activated partial thromboplastin time (s)	24.8	24.0-34.0
International normalized ratio	0.99	-
Lactate, serum (mmol/L)	3.16	0.50-2.20
Lipase, serum (U/L)	>15,000	10-60
Total cholesterol, serum (mmol/L)	2.93	0.00-5.20
Triglycerides, serum (mmol/L)	1.94	0.00-2.20

**Figure 1:**
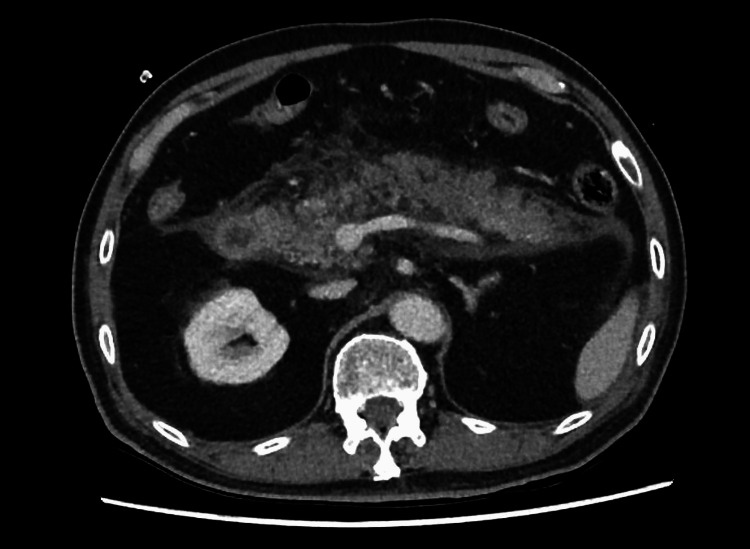
Contrast-enhanced CT in the portal venous phase, done on admission, showing hypoenhancing regions in the pancreatic neck and body

The patient’s severity stratification according to the modified Glasgow-Imrie score [3] was 3, indicating severe pancreatitis. A contrast-enhanced computed tomography (CT) was performed to assess for complications of acute pancreatitis. It showed hypoenhancing regions in the pancreatic neck and body, suspicious for necrosis, without any extra-pancreatic collections (Figure 1). No gallstones or dilatation of the pancreatic duct or biliary tree were noted. There was no thrombosis in the portal vein and splenic artery, and both kidneys were enhancing normally.

**Figure 2:**
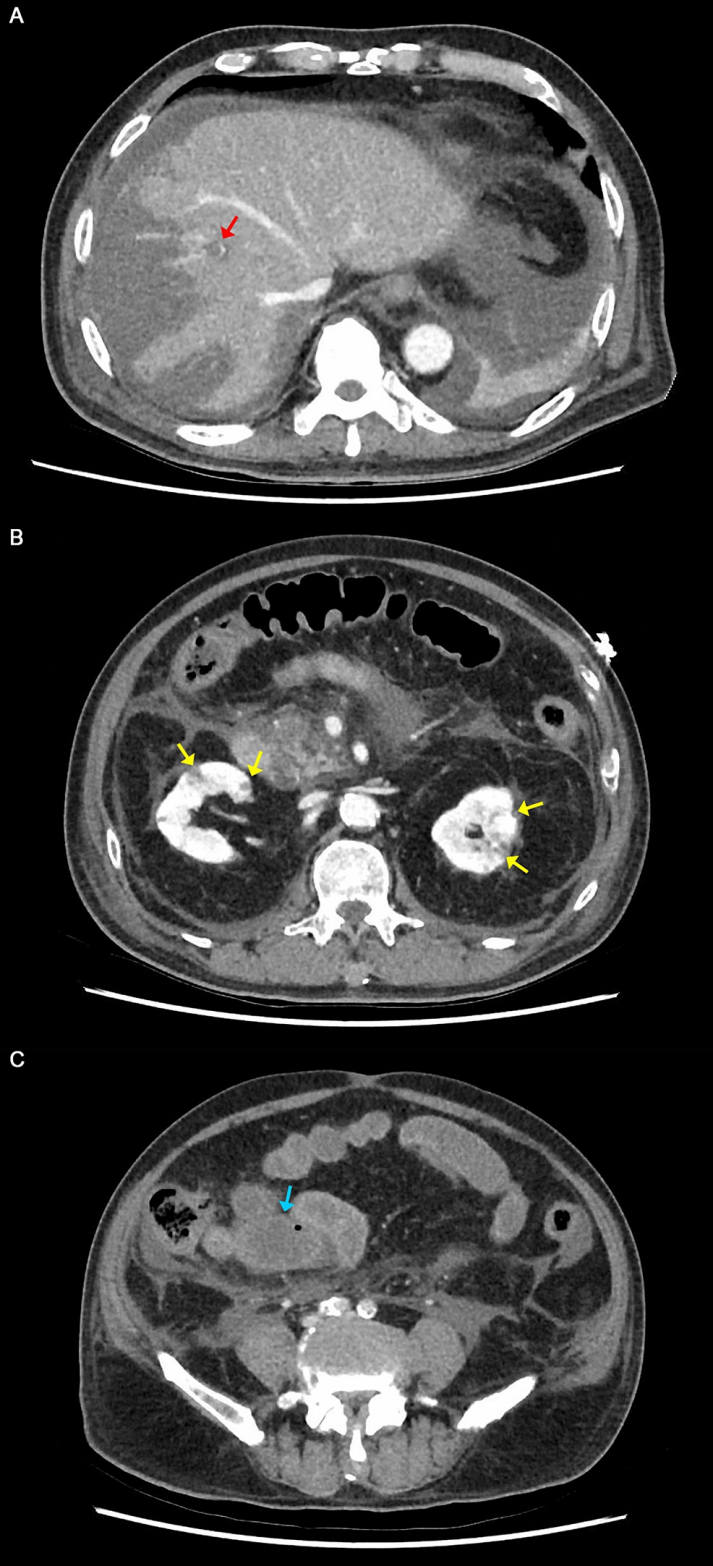
Contrast-enhanced CT in the portal venous phase done one day after admission (A) Thrombosis of the anterior segmental division of the distal right branch portal vein (red arrow) with multi-focal wedge-shaped hepatic infarcts in the right hepatic lobe. (B) Multiple peripheral wedge-shaped renal infarcts in bilateral kidneys (yellow arrows). (C) Relative hypoenhancement in the jejunal loops (blue arrow) indicating possible acute mesenteric ischaemia.

He was admitted to the surgical high dependency unit, and treated conservatively with intravenous (IV) fluids and antibiotics. Over the course of the day, he deteriorated, developing KDIGO 2 oligoanuric acute kidney injury, worsening liver dysfunction with transaminitis, lactaemia, and severe metabolic acidosis. The patient was then intubated, and started on inotropic support, before undergoing a repeat CT to look for ischaemic complications of acute pancreatitis. The CT showed interval development of thrombosis of the anterior division of the distal right branch portal vein with hepatic infarctions (Figure 2A), and small non-occlusive thrombi in the main portal vein and splenic vein. There were also multiple renal infarctions bilaterally but with patent renal arteries (Figure 2B), and possible bowel ischaemia involving the jejunum and ileum but with the patent superior mesenteric artery (Figure 2C).

The patient underwent diagnostic laparoscopy, with a view to possible laparotomy and bowel resection. Segments of the small bowel appeared unhealthy, hypoperfused, and mildly dilated, but there was no intestinal infarction or necrosis, thus no surgical resection was performed. In total, 1000 ml of haemorrhagic ascitic fluid was noted and drained from the peritoneal cavity. Post-operatively, he was transferred to the intensive care unit (ICU) for multi-organ support. He required high-dose inotropic support (noradrenaline up to 0.5 mcg/kg/min and vasopressin 0.03 units/min), ventilatory support, and continuous renal replacement therapy. IV unfractionated heparin infusion was initiated in view of the multi-focal thrombi and multiple organ infarctions present, titrated to a target activated partial thromboplastin time of 50-70 seconds. Despite treatment, he continued to deteriorate into multiple organ failure and died 36 hours later. The patient’s laboratory data over the course of his hospitalization have been summarized in Table 2.

**Table 2:** Selected laboratory data over the course of hospitalization demonstrating the patient’s clinical deterioration ^#^ Patient on continuous renal replacement therapy. ^^^ Patient on IV unfractionated heparin infusion.

Variable	Day 1 (admission)	Day 2 (immediate pre-operative)	Day 3 (24 hours post-operative)	Reference range
Leukocyte count (x 10^3^/µL)	23.2	22.4	11.2	4.0-10.0
Platelet count (x 10^3^/µL)	239	209	118	150-450
Urea, serum (mmol/L)	6.5	11.7	6.2^#^	2.8-7.7
Creatinine, serum (mmol/L)	120	285	189^#^	65-125
Total bilirubin, serum (µmol/L)	9.4	23.4	57.2	5.0-30.0
Alanine transaminase, serum (U/L)	18	1325	3856	10-55
Aspartate transaminase, serum (U/L)	21	2135	6957	10-45
Prothrombin time (s)	10.5	14.3	20.0	9.5-11.5
Activated partial thromboplastin time (s)	24.8	35.7	72.4^^^	24.0-34.0
International normalized ratio	0.99	1.41	2.08	-
pH	7.381	7.213	7.097	7.350-7.450
Bicarbonate, serum (mmol/L)	21	15	8	19-31
Lactate, serum (mmol/L)	3.16	9.74	17.70	0.50-2.20

## Discussion

SVT is a serious complication of acute pancreatitis, occurring in 16.6-22.6% of patients [4,5]. It is associated with more severe forms of the disease, particularly necrotizing pancreatitis, and its development is associated with increased morbidity and mortality [6,7]. Sequelae of untreated SVT include the development of portal hypertension with oesophageal or gastric varices, gastrointestinal bleeding, hypersplenism with anaemia and thrombocytopaenia, and intestinal ischaemia [8]. In patients with isolated splenic vein thrombosis, treatment may be conservative with close monitoring of the thrombus for recanalization. However, in patients with more extensive SVT extending to the mesenteric vein and with clinical presentation of intestinal ischaemia, such as our patient, initiation of antithrombotic therapy is recommended [8]. In our patient, IV unfractionated heparin infusion was used, as he was in acute renal failure, requiring continuous renal replacement therapy.

In acute pancreatitis, intra-acinar activation of proteolytic enzymes causes (a) direct endothelial injury, resulting in microcirculatory changes including vasospasm and capillary stasis [9], and (b) the initiation of an inflammatory response, with leukocyte chemoattraction [10] and release of pro-inflammatory cytokines such as interleukin (IL)-6, IL-8, IL-1β, tumour necrosis factor (TNF)-α, and macrophage migration inhibitory factor [10,11]. The inflammatory mediators then activate the coagulation pathway, which in turn further stimulates the inflammatory pathway, creating a positive feedback loop [10]. Furthermore, pancreatic enlargement and/or pseudocyst may directly compress the splanchnic vein, causing venous stasis [8]. Together, the trifecta of stasis, hypercoagulability, and endothelial injury, create an environment favourable for thrombosis.

COVID-19 primarily affects the respiratory system, but can also have several extrapulmonary manifestations. An association between COVID-19 infection and pancreatic injury or acute pancreatitis has been proposed, but two systematic reviews examining the relationship between COVID-19 infection and acute pancreatitis have found that there is insufficient evidence to establish a causal relationship between the two [12,13]. The association between COVID-19 infection and thromboembolism, on the other hand, is well-established, with a prevalence of >20% [14]. The hypercoagulable state induced by COVID-19 infection is thought to be due to a hyperinflammatory response to infection, which, together with hypoxia and direct viral-mediated effects on endothelial cells, may contribute to high rates of macro- and microthrombotic events [1,15].

Moreover, these prothrombotic changes appear to be sustained even after recovery from the initial COVID-19 infection, with one study finding elevated factor VIII and plasminogen-activator inhibitor type 1 levels at four months post-recovery [16]. The authors of the study postulate that these sustained changes may contribute to thromboembolic events in COVID-19 patients after recovery from the infection. In addition, COVID-19 patients who suffer thromboembolic events are at a higher risk of death compared to those without thromboembolic events [14,15]. Thus, it is recommended that hospitalized COVID-19 patients receive prophylactic anticoagulation to reduce all-cause mortality [17,18]. In critically ill patients, the risk of thrombotic events should be weighed against the risk of haemorrhagic complications before anticoagulation is initiated [17,18]. Unfortunately, there are no similar studies conducted on patients with recent COVID-19 infection who are hospitalized for other reasons, such as our patient.

We report a case of acute necrotizing pancreatitis that was complicated by multiple splanchnic thromboses resulting in multiple hepatic infarctions and jejunal hypoperfusion, as well as multiple bilateral renal infarctions. To our best knowledge, such extensive thromboses, as a complication of acute pancreatitis, have never been reported before. We postulate that the patient’s recent COVID-19 infection had led to a persistent hypercoagulable state, which, combined with the additional inflammatory response caused by acute pancreatitis, created a perfect environment for the formation of multiple thrombi. Interestingly, while multiple bilateral renal infarctions were seen on the CT scan, no renal arterial or venous thrombi were seen on the scan or during his surgery. There were also more areas of hepatic infarction than could be accounted for by the distal right branch portal vein thrombus seen on the CT scan. This could be the result of capillary microthrombi in the renal and hepatic circulation, similar to that found in the lungs of patients who had died of COVID-19 [1]. However, as this patient did not have an autopsy performed, we are unable to confirm this postulation.

A possible differential diagnosis for the hypercoagulable state observed is disseminated intravascular coagulation (DIC) caused by severe sepsis. DIC is characterized by thrombocytopaenia, prolonged prothrombin time, increased D-dimer levels, and hypofibrinogenaemia [19]. Compared to DIC, COVID-19 coagulopathy is associated with less severe thrombocytopaenia, normal or only mildly prolonged prothrombin time, and markedly elevated D-dimer and fibrinogen levels [20]. Our patient’s laboratory tests are more in keeping with COVID-19 coagulopathy, as he had no thrombocytopaenia and only mildly elevated prothrombin time at the time of his clinical deterioration on the second day of admission (Table 2). Unfortunately, D-dimer and fibrinogen levels were not measured in this patient, as clinical suspicion of DIC was low; he did not have any abnormal bleeding from his vascular lines or surgical drains despite systemic anticoagulation, nor skin manifestations such as petechiae or purpura fulminans. In hindsight, measurement of D-dimer and fibrinogen levels would have been instructive; however, the clinical manifestation of his coagulopathy was thrombotic rather than haemorrhagic, in keeping with that seen in COVID-19 coagulopathy [20].

Ideally, we would have liked to perform a thrombophilic screen for this patient to rule out other causes of his extensive thromboses. However, due to his rapid deterioration and need for surgical intervention, followed by the establishment of a do not resuscitate order after his condition further worsened post-surgery, we were unable to evaluate him further for other thrombophilic conditions. At the same time, given his advanced age with a lack of prior history of thromboembolic events, we believe that it is less likely that he had an underlying thrombophilic condition, and the most likely cause of the hypercoagulable state observed is due to the concurrent, potentiating inflammatory effects of recent COVID-19 infection and severe acute pancreatitis.

## Conclusions

COVID-19 can cause sustained prothrombotic changes that put patients at risk of thromboembolic events for several months after recovery from the infection. Acute pancreatitis also produces an inflammatory response that forms a positive feedback loop with the coagulation pathway. We believe that together, they may have resulted in the particularly extensive intra-abdominal thromboses and infarctions seen in this patient.

Physicians should be mindful of the elevated risk of thromboembolic complications in patients with recent COVID-19 infection, who are hospitalized for reasons unrelated to their infection, especially inflammatory conditions such as acute pancreatitis. There is currently no guidance regarding the use of prophylactic antithrombotics in this group of patients and this is an area that warrants further study.
